# Rapid Accumulation of Proline Enhances Salinity Tolerance in Australian Wild Rice *Oryza australiensis* Domin

**DOI:** 10.3390/plants10102044

**Published:** 2021-09-28

**Authors:** Ha Thi Thuy Nguyen, Sudipta Das Bhowmik, Hao Long, Yen Cheng, Sagadevan Mundree, Linh Thi My Hoang

**Affiliations:** Centre for Agriculture and the Bioeconomy, Queensland University of Technology (QUT), 2 George Street, Brisbane, QLD 4000, Australia; sudipta.dasbhowmik@qut.edu.au (S.D.B.); h1.long@qut.edu.au (H.L.); alam.cheng@qut.edu.au (Y.C.); sagadevan.mundree@qut.edu.au (S.M.)

**Keywords:** *Oryza australiensis*, osmotic adjustment, proline, physiological parameters, qRT-PCR, salinity tolerance, wild rice

## Abstract

Proline has been reported to play an important role in helping plants cope with several stresses, including salinity. This study investigates the relationship between proline accumulation and salt tolerance in an accession of Australian wild rice *Oryza australiensis* Domin using morphological, physiological, and molecular assessments. Seedlings of *O. australiensis* wild rice accession JC 2304 and two other cultivated rice *Oryza sativa* L. cultivars, Nipponbare (salt-sensitive), and Pokkali (salt-tolerant), were screened at 150 mM NaCl for 14 days. The results showed that *O. australiensis* was able to rapidly accumulate free proline and lower osmotic potential at a very early stage of salt stress compared to cultivated rice. The qRT-PCR result revealed that *O. australiensis* wild rice JC 2304 activated proline synthesis genes *OsP5CS1, OsP5CS2,* and *OsP5CR* and depressed the expression of proline degradation gene *OsProDH* as early as 1 h after exposure to salinity stress. Wild rice *O. australiensis* and Pokkali maintained their relative water content and cell membrane integrity during exposure to salinity stress, while the salt-sensitive Nipponbare failed to do so. An analysis of the sodium and potassium contents suggested that *O. australiensis* wild rice JC 2304 adapted to ionic stress caused by salinity by maintaining a low Na^+^ content and low Na^+^/K^+^ ratio in the shoots and roots. This demonstrates that *O. australiensis* wild rice may use a rapid accumulation of free proline as a strategy to cope with salinity stress.

## 1. Introduction

Salinity causes major constraints in global crop production [1]. The impacts of salinity on crop yield vary greatly in different plants. For example, when grown on salt-affected land in the Indian Indo-Gangetic Basin, the yields lost for rice, wheat (*Triticum aestivum* L.), cotton (*Gossypium herbaceum* L.), and sugar cane (*Saccharum officinarum* L.) were recorded at 45 %, 39 %, 63 %, and 48 %, respectively [2]. Cultivated rice *Oryza* *sativa* L. is one of the most important crops and serves more than one-half of the world’s population [3]. However, cultivated rice is also reported as one of the most salt-sensitive cereals [4]. It has been estimated that salinity results in a 20% loss of global rice production [5]. Several strategies have been used to improve salt tolerance in cultivated rice *O. sativa*, including conventional breeding, genetic modification, and genome editing [6,7].

Wild *Oryza spp.* rice has been used as a genetic resource to improve salinity stress tolerance in cultivated rice through conventional breeding for decades [8]. However, there is still a knowledge gap in understanding the salt tolerance mechanisms in these wild rice species [9]. *Oryza australiensis* Domin is an endemic wild rice species that belongs to the EE genome taxa of the *Oryza* genus. This wild rice species is perennially distributed across Northern Australia. It grows in seasonally wet locations and has a rhizome that allows the plants to survive in the dry season. Due to these characteristics, *O. australiensis* has been suggested as a potential genetic source for drought tolerance [10]. In addition, this species also has traits associated with other abiotic and biotic stress tolerance, including heat tolerance [11] and brown planthopper (*Nilaparvata lugens* Stål) resistance [12]. Recently, Yichie et al. [13] found that proteins belonging to the transport, metabolic process, and transmembrane transporter categories were highly responsive to salt treatment in *O. australiensis*. This wild rice also showed the least reduction in biomass accumulation, SES score, chlorophyll content, and lowest average shoot Na^+^/K^+^ ratio in response to salinity compared to other wild and cultivated rice under 80 mM NaCl treatment [13]. However, no study on the responses of this wild rice species to higher salt concentration has been reported.

High salt concentrations in the soil lead to deleterious effects on plants due to osmotic and ionic stresses. The short-term exposure to salinity stress resulted in the reduction of osmotic potential on the outside roots, disrupting the water uptake, leading to a significant decline in the shoot growth rate [4]. The adverse effects of osmotic stress reduce cell expansion in root tips and leaf expansion, causing stomatal closure. Consequently, it causes a decrease in photosynthesis, leading to the accumulation of reactive oxygen species (ROS) [14]. To adapt to osmotic stress, plants evolve an osmoprotectant mechanism to adjust cellular osmotic potential [15]. Under this mechanism, plants accumulate sufficient compatible osmolytes to adjust osmotic potential and balance soil osmotic pressure [16,17,18].

Proline is a proteinogenic amino acid that has been accumulated as a beneficial solute in plants under both non-stress and stress conditions [19]. Proline was first reported to be accumulated in ryegrass (*Lolium perenne* L.) upon wilting conditions [20]. The accumulation of proline naturally occurs in the cytoplasm, where it acts as an osmoprotectant to defend against osmotic stress [21,22,23,24]. A high level of proline in the cytosol reduces the cellular water potential below the external water potential, enhancing the water flow into the cells to maintain cellular water status and plant cell turgidity [22,25]. Apart from acting as an osmolyte for osmotic adjustment, proline contributes to stabilizing subcellular structures (e.g., proteins and membranes) [26], buffering cellular redox potential against stresses [22,27,28]. Proline has also been reported to play a role in mitigating ROS’s deleterious effects [27,29], maintaining the NADP/NADPH ratio compatibility and alleviating cytoplasmic pH [22]. Upon stress, proline supports mitochondrial oxidative phosphorylation to generate energy for plants to recover from stress [22,30,31]. In higher plants, proline is synthesized via glutamate or ornithine pathways [24,32]. The radioisotope labeling experiment indicated that the glutamate pathway is predominant for proline synthesis during osmotic stress. In contrast, the ornithine pathway was the primary route under high nitrogen application [24]. In the glutamate pathway, glutamate is converted to γ-glutamyl semialdehyde (GSA) by a bifunctional enzyme Δ-pyrroline-5-carboxylate synthetase enzyme (P5CS), which has both γ-GK and γ-GPR domains [33]. GSA then spontaneously cyclizes to Δ-pyrroline-5-carboxylate (P5C). P5C is then reduced to proline by enzyme Δ-pyrroline-5-carboxylate reductase (P5CR) [17,26]. Proline dehydrogenase (ProDH) catalyzes proline to P5C in mitochondria [26]. The proline content was reported to increase in many plants during salinity stress [34,35,36]. The accumulation of proline occurs under salinity stress mainly due to the stimulated synthesis in the tissue, the inhibition of proline oxidation in the mitochondria, and the plant’s ability to maintain the mitochondria membrane’s permeability [24,37].

This study investigates the accumulation of free proline in *O. australiensis* accession JC 2304 during the exposure to high salinity stress and analyses the relationship between proline accumulation and salt tolerance in this wild rice species using molecular, morphological, and physiological assessments.

## 2. Results

### 2.1. Wild Rice O. australiensis Rapidly Accumulated Free Proline under Salinity Stress

**Figure 1:**
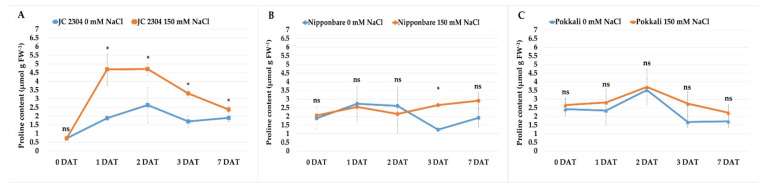
Effects of salinity stress (150 mM NaCl) on free proline content of wild and cultivated rice at the seedling stage. (**A**) Wild rice *O. australiensis* JC 2304. (**B**) Cultivated rice *O. sativa* cultivar Nipponbare. (**C**) Cultivated rice *O. sativa* cultivar Pokkali. Data are means ± SE (*n* = 3). Asterisks indicate the significant difference (*p ≤* 0.05), ns-not significant, based on the Student *t*-test between salt-stressed and non-stressed counterparts. DAT—day after treatment. FW—fresh weight.

Proline is one of the most common osmolytes that contributes to the cellular osmotic potential change under various abiotic stresses. In this study, we observed that *O. australiensis* wild rice accession JC 2304 accumulated higher free proline content and more rapidly in response to salinity stress compared to the salt-tolerant (Pokkali) and salt-sensitive (Nipponbare) *O. sativa* cultivars (Figure 1). Before the onset of salinity stress, free proline contents in cultivated rice cultivars were around 2 μmol g^−1^ FW (Figure 1B,C); meanwhile, the wild rice had only 0.71 μmol g^−1^ FW free proline (Figure 1A). However, within 48 h of salinity stress, a clear contrasting difference in the proline content was observed between the wild rice and the cultivated rice cultivars. Wild rice JC 2304 sharply increased free proline content nearly seven times, from 0.71 μmol g^−1^ FW to 4.68 μmol g^−1^ FW, after 24 h of exposure to salinity stress and steadily maintained at 4.72 μmol g^−1^ FW for up to 48 h (Figure 1A). In contrast, the two cultivated rice cultivars, Nipponbare and Pokkali, showed slight increases in free proline content by only 0.09 and 0.52 μmol g^−1^ FW, respectively (Figure 1B,C). After 72 h of salinity stress exposure, the proline content in the *O. australiensis* wild rice declined to about 3.27 μmol g^−1^ FW; meanwhile, Nipponbare and Pokkali had lower levels of proline content. The Nipponbare salt-stressed plants had a slight increase in free proline content to 3.0 μmol g^−1^ FW at day seven of salt stress, whereas the salt-stressed plants of Pokkali had a slight decrease in proline content to 2.0 μmol g^−1^ FW. Wild rice *O. australiensis* still maintained a high free proline content at 2.5 μmol g^−1^ FW. Overall, wild rice JC 2304 and Pokkali showed the same trend in proline accumulation, i.e., more proline was accumulated in the leaves of salt-stressed plants compared to non-stressed plants. However, the significant proline accumulation was only observed in JC 2304 but not Pokkali during the seven days of testing (Figure 1A,C). Nipponbare, on the other hand, showed the opposite trend to JC 2304 and Pokkali. This cultivar did not accumulate proline in the first 48 h of exposure to NaCl treatment but later at three and seven DAT (Figure 1B). These results suggest that *O. australiensis* wild rice may rapidly accumulate proline to withstand high salinity stress. 

### 2.2. Wild Rice O. australiensis Activated Proline Synthesis and Depressed Proline Degradation Genes at the Early Stage of Exposure to Salinity Stress

**Figure 2:**
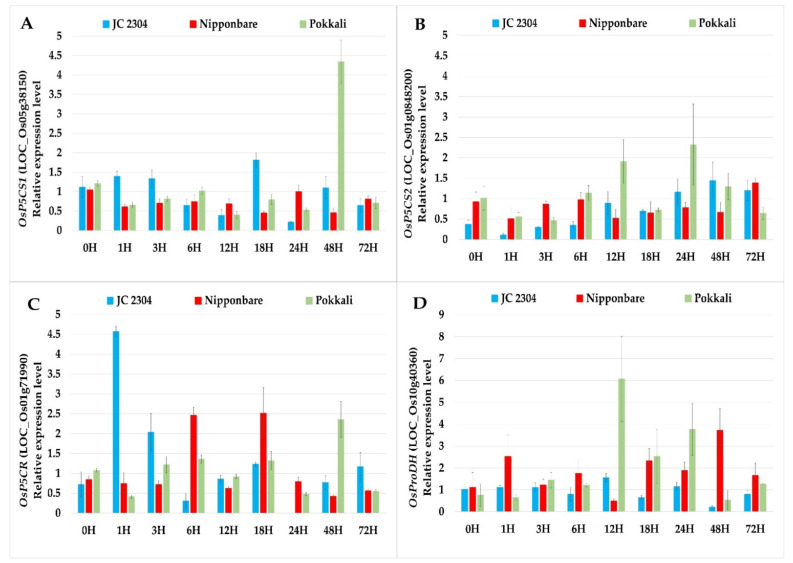
Effects of salinity stress (150 mM NaCL) on relative expression of proline metabolism genes in wild and cultivated rice. The relative expression was quantified as fold change in leaf tissue of cultivated rice (*O. sativa*) and wild rice (*O. australiensis*) by quantitative Real-Time PCR (qRT-PCR). (**A**) *OsP5CS1*. (**B**) *OsP5CS2*. (**C**) *OsP5CR*. (**D**) *OsProDH*. Levels of the transcript were normalized to two internal reference genes *OsAct* and *OsEF-1α.* Expression levels of genes in salt-stressed plants were normalized with respect to those in non-stressed plants. Data represent the means ± SE (*n* = 9). H-hours.

The results of the qRT-PCR gene expression analyses showed that the genes related to proline biosynthesis (*OsP5CS1* and *OsP5CR*) were upregulated in *O. australiensis* wild rice JC 2304 as early as 1 h upon exposure to 150 mM NaCl. The transcripts levels of *OsP5CS1* and *OsP5CR* in leaves of *O. australiensis* wild rice were observed to increase 1.5- and 4.5-fold within the first hour of salinity exposure, respectively (Figure 2A,C). On the contrary, none of the genes involved in proline biosynthesis were upregulated in Nipponbare or Pokkali before the 6 h time point. Noticeably, the expressions of two *OsP5CS* genes in Nipponbare were not switched on until 72 h of salinity stress (Figure 2A,B). Another proline synthesis gene, *OsP5CR,* was upregulated approximately 2.5-fold at 6 h and 18 h but remained downregulated at all other time points in Nipponbare. In Pokkali, the expression level of the *OsP5CS2* gene increased significantly at 12 h and 24 h following the salinity treatment; however, the other genes in the proline biosynthesis pathway, *OsP5CS1* and *OsP5CR,* were significantly upregulated only after 48 h of salt exposure (Figure 2). In terms of the proline degradation gene, we observed a 2.5-fold increase in the expression level of *OsProDH* in Nipponbare as early as 1 h after the salinity treatment, and the highest transcripts level of this gene in Nipponbare was recorded at the 48 h time point with a 3.7-fold increase (Figure 2D). In Pokkali, the transcripts level of *OsProDH* peaked at 12 h after salt stress with an approximately 6.1-fold increase, and the expression level decreased thereafter. In contrast, no change was noticed in the transcripts level of *OsProDH* in *O. australiensis* wild rice JC 2304 during a 72 h exposure to salinity stress except for a noticeable downregulation at the 48 h time point (Figure 2D).

### 2.3. Wild Rice O. australiensis Showed a High Tolerant Level to Salinity Stress

The salinity tolerance of each rice genotype was evaluated based on the salt damage symptoms of the leaves and plants. In our study, we used the standard evaluation score (SES) of visual salt injury developed by Gregorio et al. at the International Rice Research Institute (IRRI) [38]. We observed that *O. australiensis* wild rice accession JC 2304 showed a similar level of tolerance to salinity stress to the well-known salt-tolerant cultivar Pokkali and a greater tolerance than the salt-sensitive cultivar Nipponbare. 

**Figure 3:**
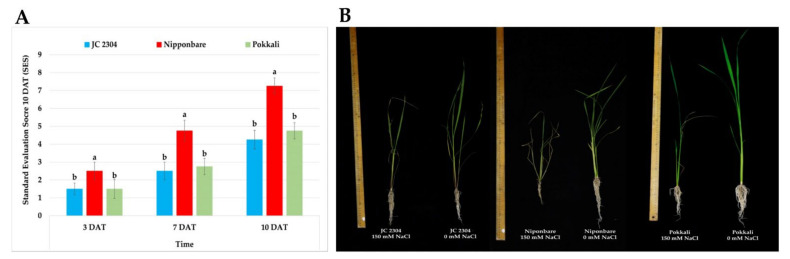
Salinity tolerance of wild and cultivated rice under 150 mM NaCl treatment. (**A**) SES of wild rice JC 2304 and two cultivated rice cultivars (salt-sensitive cultivar Nipponbare and salt-tolerant cultivar Pokkali). (**B**) Phenotypes of wild and cultivated rice under 150 mM NaCl treatment for 10 days. Data represent the means ± SE (*n* = 8). Values labelled with different letters are significantly different at *p <* 0.05, based on the Turkey HSD test. DAT—day after treatment.

On day three of exposure to 150 mM NaCl stress, the wild rice exhibited a similar SES to the salt-tolerant rice Pokkali (SES = 1.5 ± 0.32). The salt-sensitive cultivar Nipponbare, in contrast, started to show mild symptoms of salt injury, with an SES around 2.50 ± 0.50. With prolonged stress exposure (seven DAT), *O. australiensis* wild rice accession JC 2304 (SES = 2.5 ± 0.5) was still ranked as similarly tolerant compared to Pokkali (SES = 2.75 ± 0.45), while Nipponbare had an SES of 4.75 ± 0.59. As salt stress continued to 10 days, *O. australiensis* wild rice JC 2304, with an SES of 4.25 ± 0.52, was rated to be as tolerant as Pokkali (SES = 4.75 ± 0.45) (Figure 3A). On the other hand, the Nipponbare cultivar was rated as salt-sensitive to 150 mM NaCl and showed severe salt damage symptoms with an SES around 7.25 ± 0.45. Regarding phenotypic changes, the wild rice and Pokkali salt-stressed plants showed less salt injury symptoms, such as chlorosis or leaf rolling, compared to Nipponbare, which exhibited wilted leaf symptoms on day 10 of salt treatment (Figure 3B).

### 2.4. Wild Rice O. australiensis Exhibited Higher Root Growth and Biomass Than Cultivated Rice under Salinity Stress

**Figure 4:**
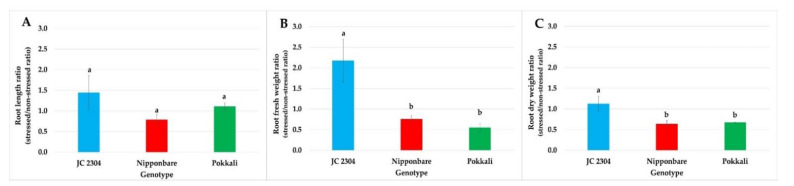
Effects of salinity stress (150 mM NaCl) on root length and root biomass (14 DAT) of wild and cultivated rice at the seedling stage. (**A**) Relative root length. (**B**) Relative root fresh weight. (**C**) Relative root dry weight. Data represent the means ± SE (*n* = 4). Value labeled with different letters is significantly different at *p <* 0.05, based on the Turkey HSD test.

Increasing salt concentration in soils causes osmotic stress that affects cell expansion in root tips and, consequently, reduces root growth. Therefore, in this study, the effects of salinity stress on the root growth and biomass of *O. australiensis* wild rice JC 2304 and cultivated rice Nipponbare and Pokkali were investigated. The root length, fresh weight (FW), and dry weight (DW) data of salt-stressed plants on day 14 were compared to those of their respective non-stressed counterparts as the mean of ratio (Figure 4). As shown in Figure 4A, the relative root length of *O. australiensis* wild rice accession JC 2304 increased by 44% (stressed/non-stressed root length ratio ~1.44), while the salt-sensitive cultivated rice Nipponbare declined in root length by 22% (stressed/non-stressed root length ratio ~0.78). No change was observed in the root length of the Pokkali plants under stressed and non-stressed conditions (Figure 4A). On measuring the root FW ratio, *O. australiensis* wild rice JC 2304 significantly maintained a higher relative root fresh weight by more than two-fold than Nipponbare and Pokkali, which had 25% and 46% reduced root fresh weights, respectively (Figure 4B). In addition, wild rice JC 2304 maintained a significantly higher relative root dry weight in comparison to the two cultivated rice, Nipponbare and Pokkali (Figure 4C).

### 2.5. Wild Rice O. australiensis Lowered Osmotic Potential at the Early Stage of Exposure to Salinity Stress

**Figure 5:**
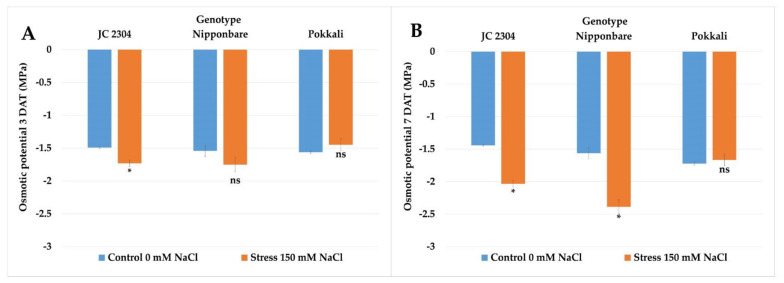
Effects of 150 mM NaCl on the osmotic potential of seedlings of wild and cultivated rice. (**A**) Osmotic potential 3 DAT. (**B**) Osmotic potential 7 DAT. Data represent means ± SE (*n* = 3). Asterisks indicate the significant difference of salt-stressed plants with respect to non-stressed plants, based on the Student *t*-test (ns-not significant, * *p ≤* 0.05). DAT—day after treatment.

To determine whether the rapid accumulation of free proline in *O. australiensis* affects osmotic potential in this wild rice under salinity stress, we measured the osmotic potential in the second youngest fully expanded leaves of the wild rice and cultivated the rice on days three and seven following the salt treatment. The results showed that *O. australiensis* wild rice JC 2304 significantly lowered the osmotic potential from −1.49 MPa to −1.73 Mpa at day three of salt stress (Figure 5). Cultivated rice cultivars, on the contrary, had no significant decreases in osmotic potential at three DAT (Figure 5A). Furthermore, the osmotic potential in the Nipponbare cultivar started to decrease significantly from day seven of salt stress, while the Pokkali cultivar still maintained an unchanged osmotic potential, whereas *O. australiensis* wild rice JC 2304 recorded an approximately 40% decrease in osmotic potential compared to the non-stressed controls (Figure 5B). These results suggest that proline accumulation in the *O. australiensis* wild rice JC 2304 might contribute to adjusting osmotic potential lowering in this wild rice accession.

### 2.6. Wild Rice O. australiensis Maintained Higher Relative Water Content and Cell Membrane Integrity under Salinity Stress Compared to Salt-Sensitive Cultivated Rice

**Figure 6:**
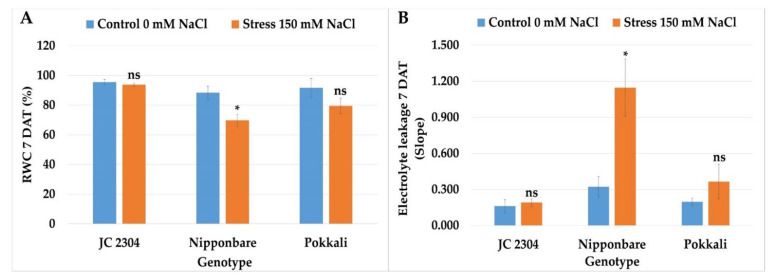
Effects of 150 mM NaCl on relative water content and electrolyte leakage of wild and cultivated rice at 7 days exposure to salinity stress. (**A**) RWC. (**B**) Electrolyte leakage. Data represent the means ± SE (*n* = 3). Asterisks indicate the significant difference of salt-stressed plants with respect to non-stressed plants, based on the Student *t*-test (ns-not significant, * *p ≤* 0.05). DAT—day after treatment.

Relative water content (RWC) and electrolyte leakage were used in many studies as key physiological parameters to evaluate salinity tolerance in rice [39,40,41]. Therefore, in this study, we measured the RWC and electrolyte leakage in *O. australiensis* wild rice JC 2304, Pokkali, and Nipponbare plants’ exposure to 150 mM NaCl at day seven of salt-treatment. Figure 6A shows that *O. australiensis* wild rice JC 2304 maintained the RWC at day seven at about 93.79%, which was not significantly different from the non-stressed control counterparts and higher than was observed in the stressed Pokkali plants. On the contrary, the salt-sensitive cultivated rice Nipponbare had a significant reduction in RWC by about 30% (Figure 6A). In terms of electrolyte leakage, Nipponbare encountered a sharp increase in electrolyte leakage by 3.8 times compared to the non-stressed controls after seven days of exposure to salinity stress. In contrast, the electrolyte leakage of both *O. australiensis* wild rice accession JC 2304 and the salt-tolerant Pokkali exhibited no significant increase compared to the respective non-stressed control counterparts (Figure 6B). These results further demonstrated that *O. australiensis* wild rice and the salt-tolerant cultivated rice Pokkali have abilities to mitigate the adverse effects of salinity on RWC and maintain plasma membrane integrity.

### 2.7. Wild Rice O. australiensis Accumulated Less Toxic Ion and Maintained a Lower Na^+^/K^+^ Ratio in Shoots under Salinity Stress Compared to Cultivated Rice

**Figure 7:**
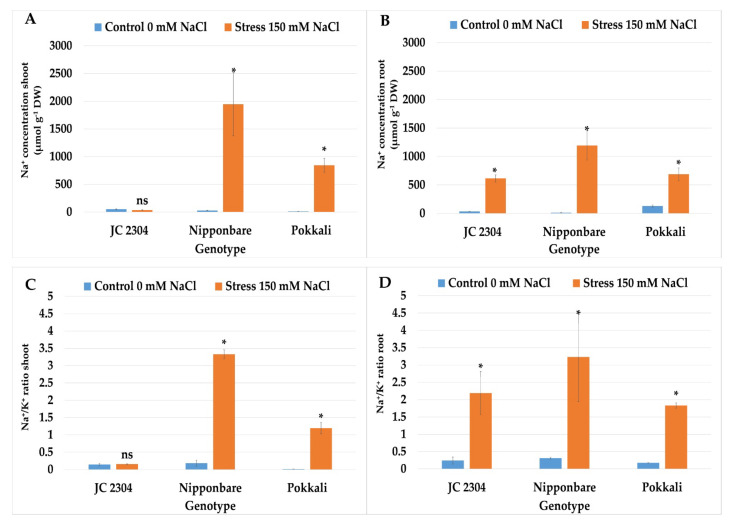
Effects of 150 mM NaCl on sodium and potassium contents in wild and cultivated rice seedlings after 14 days of stress. (**A**) Sodium content in shoots (**B**) Sodium content in roots. (**C**) Potassium content in shoots (**D**) Potassium content in roots. Data represent the means ± SE (*n* = 9). Asterisks indicate the significant difference of salt-stressed plants with respect to non-stressed plants, based on the Student *t*-test (ns-not significant, * *p ≤* 0.05). DW—dry weight.

An excessive concentration of salinity in soil induces the accumulation of toxic ions in the roots and shoots of plants, injuring cells in transpiring leaves, leading to plant growth reduction. The accumulation of sodium ions in plant cells constrains potassium ions’ uptake, which is essential for plant growth. Therefore, the sodium and potassium ion content were measured in the shoots and roots of salt-stressed plants at 14 days post-salinity treatment. Under non-stressed conditions, all three of the rice genotypes maintained the minimum Na^+^ in their shoots (Figure 7A). However, under 150 mM NaCl treatment, a rapid increase in Na^+^ concentration was observed in the shoots of Nipponbare and Pokkali genotypes. Nipponbare accumulated 1946.68 μmol g^−1^ DW Na^+^, whereas Pokkali accumulated 843.2 μmol g^−1^ DW Na^+^ in the shoots, which was significantly higher than those in the non-stressed control counterparts (Figure 7A). On the contrary, *O. australiensis* wild rice accession JC 2304 accumulated only 34.67 μmol g^−1^ DW Na^+^ in the shoots, which was not significantly different compared to the non-stressed control plants (Figure 7A). The roots accumulated significantly more Na^+^ in all three of the tested genotypes compared to their respective non-stressed controls. Among these three genotypes, *O. australiensis* wild rice accession JC 2304 accumulated the least sodium content (613.6 μmol g^−1^ DW), whereas Nipponbare plants accumulated the most (1193.51 μmol g^−1^ DW) in their roots (Figure 7B). The relative Na^+^/K^+^ ratio in the shoots of non-stressed plants of all the genotypes exhibited less accumulation of Na^+^ ion than K^+^ ion. However, salinity stress induced significant increases in Na^+^ accumulation in the shoots of stressed Nipponbare and Pokkali plants. In contrast, *O. australiensis* wild rice JC 2304 did not significantly increase the Na^+^/K^+^ ratio in the shoots compared to the respective non-stressed control counterparts (Figure 7C). Wild rice *O. australiensis* JC 2304 showed the lowest accumulation of Na^+^ in the shoots, with a Na^+^/K^+^ ratio of 0.15, whereas the concentration of Na^+^ accumulations in the shoots of salt-stressed plants of Nipponbare were 3.3 times higher than those in non-stressed plants (Figure 7C). Like the shoots, the relative Na^+^/K^+^ ratio in the roots of all three of the genotypes under non-stressed conditions remained very low (Figure 7D). Under salinity stress, *O. australiensis* wild rice JC 2304 and the salt-tolerant cultivated rice Pokkali maintained a lower Na^+^/K^+^ ratio in their roots than in the salt-sensitive cultivated rice Nipponbare. The salt-stressed plants of *O. australiensis* wild rice JC 2304 and Pokkali had Na^+^/K^+^ ratios in their roots at 2.19 and 1.82, respectively. On the contrary, the salt-sensitive cultivated rice Nipponbare plants had the highest accumulation of Na^+^ and less expulsion of K^+^ in their roots with a Na^+^/K^+^ ratio of 3.22 (Figure 7D). The results suggest that *O. australiensis* wild rice accession JC 2304 may employ a mechanism to exclude Na^+^ from the roots, thereby maintaining a low Na^+^/K^+^ ratio in the shoots.

## 3. Discussion

Salinity is a growing worldwide problem. It is one of the two most critical factors in abiotic stresses affecting crop production [15]. Efforts are ongoing to identify high salt-tolerant genetic resources for the improvement of salt tolerance in rice [6,42]. Wild rice *O. australiensis* is considered a potential candidate due to its ability to tolerate many abiotic and biotic stresses [39,43,44,45,46]. However, tolerance to high concentrations of salt, a criterion for being a salt-tolerant trait donor, has not been studied in this wild rice species. Salinity tolerance in rice is a quantitative trait that involves a complex of physiological responses, metabolic processes, and gene expression networks [47,48]. A previous study reported that salt tolerance in *O. australiensis*, exposure to 80 mM NaCl, resulted in a high response of proteins that belong to the transport, metabolic process, and transmembrane transporter categories [49]. In this study, we found that *O. australiensis* wild rice accession JC 2304 exhibited tolerance to 150 mM NaCl by rapidly accumulating free proline, lowering osmotic potential, maintaining high RWC, high membrane integrity, and low Na^+^ in the shoots and roots. 

Proline is one of the most common osmolytes that accumulate in many plants under various environmental stresses, including salinity [26,50]. The results of our investigation on proline accumulation in the leaves of wild and cultivated rice after being exposed to 150 mM NaCl treatment indicated that *O. australiensis* wild rice rapidly accumulated a high amount of free proline during the first 48 h of salt stress treatment (Figure 1A). On the other hand, the salt-tolerant cultivated rice Pokkali accumulated less free proline in the first 48 h of NaCl treatment compared to JC 2304; meanwhile, the salt-sensitive cultivar Nipponbare showed unchanged proline levels during this period of the salt treatment (Figure 1B,C). Proline accumulation under salinity stress has been reported to confer salinity tolerance in many plants, such as green gram (*Phaseolus aureus* L.) [51], wheat (*Triticum aestivum* L.) [52], mulberry (*Morus alba* L.) [53], canola (*Brassica napus* L.) [54], and Jerusalem artichoke (*Helianthus tuberosus* L.) [37]. In cultivated rice *O. sativa*, the accumulation of free proline under salt stress varies between different cultivars and growth stages. Several studies indicated that salt-tolerant rice cultivars accumulated higher proline than salt-sensitive rice under salinity-stressed conditions [55,56,57,58]. In this study, we found that the accumulation of proline, especially during the early stage (48 h) of salinity stress, positively correlated with the salt-tolerant level of *O. australiensis* wild rice JC 2304 (Figure 1A and Figure 3). We also noticed that the time of proline accumulation and the amount of this osmolyte in plants influenced their ability to cope with salt stress in the accessions tested. Both *O. australiensis* wild rice JC 2304 and Pokkali accumulated proline to a high level (above 3.5 μmol g^−1^ FW) within 48 h of exposure to 150 mM NaCl, and they showed a higher salt-tolerant level compared to Nipponbare, which did not accumulate proline during the first 48 h of salt treatment (Figure 1 and Figure 3). The salt-sensitive cultivar Nipponbare started increasing the proline content in the leaves at three DAT, but to a much lower concentration compared to that in *O. australiensis* wild rice JC 2304 and Pokkali (Figure 1). 

In higher plants, free proline accumulation results from the upregulation of proline synthesis and downregulation of proline degradation [26,59]. The results of our study agreed with these reports. Wild rice JC 2304 showed the upregulation of the genes involved in proline synthesis, including *OsP5CS1, OsP5CS2,* and *OsP5CR,* and downregulation of the gene for proline degradation, *OsProDH,* during the first 48 h exposure to salt treatment, leading to proline accumulation in these plants. The salt-tolerant cultivar Pokkali showed high expression of the genes for proline synthesis but also up-regulated the *OsProDH* gene at 12 h after salt treatment; therefore, less proline was accumulated in these plants compared to *O. australiensis* wild rice JC 2304 plants. Meanwhile, the salt-sensitive Nipponbare showed an opposite trend to the wild rice, i.e., the downregulated genes for proline synthesis and upregulated gene for proline degradation led to no proline accumulation in this cultivar in the first 48 h of salt treatment (Figure 1 and Figure 2). 

Rapid response to osmotic stress is critical for plants to maintain water uptake, thereby maintaining cellular water status and growth rate [4]. Under salinity stress, the presence of salt in the root zone triggers the osmotic stress that causes constraints on water uptake by the root system. Thus, maintaining sufficient water uptake is critical for plants to maintain cellular metabolic processes under salinity stress [60]. A positive correlation between proline accumulation and a decrease in osmotic potential was reported previously in salt-tolerant plants [51]. In our study, the lower osmotic potential in *O. australiensis* wild rice JC 2304 likely facilitated the water uptake that resulted in a higher RWC in this wild rice compared to the salt-sensitive cultivar Nipponbare (Figure 6A). These results suggest that this wild rice rapidly altered its osmotic adjustment to adapt to osmotic stress and maintain sufficient cellular water content, while the salt-sensitive cultivated rice Nipponbare showed a much later response, leading to a greater reduction in RWC and growth (Figure 4). A previous study reported that salt-tolerant plants showed less reduction in RWC than salt-sensitive plants during salinity stress [61]. In this study, the wild rice and Pokkali showed higher tolerance to salinity stress than Nipponbare (Figure 3). They also had less reduction in RWC compared to that cultivar during exposure to 150 mM NaCl treatment (Figure 6A). Interestingly, the salt-tolerant cultivar Pokkali did not show a rapid increase in free proline content but gradual accumulation, and its osmotic potentials of the salt-stressed plants were not significantly different from those of their non-stressed control counterparts (Figure 5). This observation, together with a previous report [62], indicates that stressed Pokkali plants did not employ the osmotic adjustment as a predominant mechanism to cope with salinity stress. These results led us to hypothesize that the rapid and high accumulation of proline might be a key osmolyte contributing to osmotic potential adjustment and high RWC in this wild rice accession. Osmotic stress causes the overproduction of reactive oxygen species (ROS), which, in turn, causes damage to the cell membrane [63]. The movement of salts into plant cells leads to the alternation of plasma membrane permeability [64]. Our study presents the first observation of electrolyte leakage changes in *O. australiensis* wild rice upon high salinity stress. The results of electrolyte leakage were supported by a previous study indicating that salt-tolerant genotypes maintained lower electrolyte leakage during salinity stress compared to salt-sensitive genotypes [65]. The wild rice and the salt-tolerant Pokkali maintained a low electrolyte leakage during exposure to 150 mM NaCl, whereas the salt-sensitive cultivar Nipponbare increased electrolyte leakage significantly at day seven post-NaCl treatment (Figure 6B). The maintenance of low electrolyte leakage indicated that the wild rice and Pokkali maintained plasma membrane integrity under salt stress. The ability of the wild rice and Pokkali to retain plasma membrane integrity might relate to the beneficial role of proline to either (i) mitigate the negative effects of ROS [66], (ii) sustain membrane lipid and protein compositions [67], or (iii) reduce activities of lipid peroxidation and protein oxidation [68].

Under optimal conditions, plants maintain a high concentration of K^+^ and a low concentration of Na^+^ in the cytosol. Under salt stress, the movement of Na^+^ to the roots disrupts K^+^ uptake and K^+^, Na^+^ homeostasis [25]. To overcome these conditions, plants deploy an ion homeostasis mechanism, which relies on (i) ion exclusion from the leaf and (ii) the accumulation and compartmentation of sufficient Na^+^ and Cl^-^ ions in vacuoles to balance with those in the soils [69]. While most of the halophytes employ ion compartmentation as the majority adaptive trait to ionic stress, rice is tolerant to ionic stress through ion exclusion and low Na^+^ concentrations maintenance in leaves [70]. Under salt stress, the maintenance of K^+^ and Na^+^ homeostasis are key components of salt stress tolerance in plants [71,72,73]. The results of Na^+^ and K^+^ contents and the ratio between these ions in our study indicated that the Na^+^ content in the shoots and roots of the salt-sensitive cultivated rice Nipponbare were significantly higher than those in Pokkali and the wild rice (Figure 7A,B). Interestingly, *O. australiensis* wild rice accession JC 2304 maintained much lower Na^+^ in the roots than Pokkali, which is well-known cultivated rice that employs ion homeostasis to exclude Na^+^ from the roots (Figure 7B) [74]. The molecular mechanism that helped this cultivar regulate the translocation and compartmentation of Na^+^ to the leaf tissue and vacuoles was well-documented [75,76,77]. Wild rice *O. australiensis* accession JC 2304 also has the lowest Na^+^/K^+^ ratio in the shoots and the second-lowest Na^+^/K^+^ ratio in the roots amongst the accessions tested (Figure 7C). These results suggest that, besides osmotic adjustment, *O. australiensis* wild rice employs ion homeostasis to cope with salinity stress. An investigation on the regulation of the genes involving Na^+^/K^+^ transporter and Na^+^/H^+^ exchanger in JC 2304 under salinity stress would provide details on the ion homeostasis capacity of this wild rice. The ability of roots to retain K^+^ and maintain low Na^+^/K^+^ has been reported to be critical regarding conferring salinity tolerance in cereals [78]. Therefore, further investigation on the molecular responses under this mechanism in the wild rice would provide more useful information for improving salt tolerance through ion homeostasis in cultivated rice. 

In summary, this paper details the investigation on proline accumulation and salt tolerance in wild rice *O. australiensis* and two cultivated rice *O. sativa* cultivars. The rapid accumulation of free proline in *O. australiensis* resulted in osmotic adjustment and salt tolerance in this wild rice. In addition to osmotic adjustment, wild rice *O. australiensis* also uses ion homeostasis as a mechanism to cope with salinity stress. Our study revealed a new salinity tolerant wild germplasm *O. australiensis* and the knowledge of salt tolerance mechanisms in this wild rice species would be useful for the future improvement of salt tolerance in cultivated rice. 

## 4. Materials and Methods

### 4.1. Plant Material

The wild rice seeds used in this study were obtained from the Australian Grains Genebank (AGG) (Horsham, VIC, Australia) (Supplemental Table S1). 

### 4.2. Seedling Preparation and Salinity Stress Assay

Intact wild rice seeds were heat-treated at 50 °C in an oven for three days before the experiment. Approximately 30 heat-treated seeds were then dehulled and washed 3–4 times with tap water to remove leftover hulls, followed by soaking in warm water in a 50 mL Falcon tube at 52 °C for 10 min. The Falcon tube containing seeds was then incubated in the dark at 37 °C for 48 h and finally transferred to water-soaked filter paper in a petri dish for germination. The germinated seeds were transferred to pots (10 cm diameter) containing potting mix (Searles, Australia). The seedling pots were placed inside a container and filled with tap water up to half the pot in height and kept under controlled growth conditions at 27 °C/25 °C day/night, 16 h/8 h light/dark. Salinity stress assay was conducted on three fully expanded leaves’ seedlings by adding 150 mM of saline water into the seedling containers to one cm above the potting mix level. Experiments were conducted in a Randomized Complete Block design. Salinity tolerant level of each rice genotype was evaluated based on the visual salt-induced symptoms (VSI) recorded on the 3rd, 7th, and 10th day after treatment (DAT) and converted to the modified standard evaluating score (SES) [38]. The scoring discriminates from highly susceptible (score 9) to highly tolerant (score 1). Growth rates were evaluated based on the root lengths and fresh and dry weights of roots measures at day 14 of the salinity treatment. 

### 4.3. Determination of Proline Content

Proline accumulation in leaf tissue was determined via reaction with ninhydrin (Sigma Aldrich), as described by Bates et al. [79]. Purified proline (Sigma Aldrich, Melbourne, VIC, Australia) was used to build a standard curve for proline content quantification. Approximately 0.5 fresh leaf samples were homogenized in 10 mL of 3% aqueous sulfosalicylic acid and centrifuged at 3000 rpm for 1 min. Exactly 2 mL of supernatant was reacted with 2 mL of ninhydrin acid and 2 mL of glacial acetic acid for 1 h at 100 °C in a heater. The chromophore was extracted using 2 mL of Toluene, and its absorbance at 520 nm was determined by Genesys 10-s UV/Vis Spectrophotometer (Thermo Spectronic, Waltham, MA, USA) with toluene used as blank. Proline content was calculated using the following formula.

((μg proline/mL × mL toluene)/115.5 μg/μmole) × (g sample/5) = μmoles proline gram FW^−1^

### 4.4. Gene Expression Analysis

Leaf tissues of control and salt-stressed plants were frozen in liquid nitrogen and ground to fine powder without thaw. Total RNA was extracted from 100 mg of leaf tissue by using the GeneJet Plant RNA purification kit (Thermo Scientific, Waltham, MA, USA) according to the manufacturer’s instructions. RNA was treated with RQ1 RNase-Free DNase (Promega, Alexandria, NSW, AU) to degrade any DNA contamination. The first strand of cDNA was synthesized from DNase-treated RNA using GoScript^TM^ IV first-Strand synthesis following the manufacturer’s protocols. Quantitative real-time PCR (qRT-PCR) was performed on a CFX 384™ Real-time PCR system (BioRad, Foster City, CA, USA). A 10 μL reaction mixture contained 50 ng of cDNA and 8 μL Applied Biosytems™ PowerUp™ SYBR™ Green Master Mix (Applied Biosystems™, Carlsbad, CA, USA), and 30 pmol of forward and reverse primers. Sequences of primers used in this study were presented in Supplemental Table S2. Sequences of primers for amplifying *OsP5CS1* and *OsActin* genes were acquired from a previous study [80]. Cycling conditions were as follows: 95 °C for 3 min, followed by 45 cycles at 95 °C for 10 s and 60 °C for 30 s. A melting profile was set at 95 °C for 10 s, 65 °C for 5 s, and 95 °C for 5 s. The expression levels of two internal reference genes, *OsAct1* and *OsEF1α,* were used as calibrators to calculate relative expression levels of target genes by ∆∆Ct method [81]. Three biological replicates were collected for each assessed time point, and three technical replicates were run for each biological replicate. 

### 4.5. Determination of Relative Water Content

The relative water content of plant leaf was examined using the method described by Hoang et al. [41]. Briefly, the relative water content of the leaf was measured on the second youngest fully expanded leaf. A 10 cm piece in the middle part of the leaf blade was excised, then weighed to record the fresh weight (FW). The leaf piece was then transferred to a 15 mL Falcon tube filled with distilled water and kept in the dark at 4 °C overnight. The following morning, the leaf was blotted dry with a tissue towel for 30 s and weighed to record the turgid weight (TW). The dry weight (DW) of the sample was determined after three days of drying in a vacuum oven at 70 °C. The relative water content was calculated as RWC = (FW − DW) × 100/(TW − DW).

### 4.6. Determination of Osmotic Potential

The osmotic potential was calculated based on the measurement of leaf sap osmolality (mOsm kg^−1^). Briefly, the second youngest fully expanded leaves of non-stressed and salt-stressed plants were excised and frozen at −80 °C for 12 h; cell sap was squeezed out of frozen leaf samples using a 5 mL syringe. An aliquot of 20 µL of cell sap was used for osmolarity measurement with a freezing point micro-osmometer Fiske 210 (John Morris Scientific, Chatswood, NSW, AU) at room temperature (298 °K). The osmotic potential was calculated using the following van’t Hoff equation: Π = −(M × R × T)/1000. M: molar concentration of solute in dilute solution; R: ideal gas constant (0.082); T: the room temperature on the Kelvin scale (298 °K).

### 4.7. Determination of Sodium and Potassium Content

The preparation of sample material for subsequent micronutrient analysis followed the protocols described by the Centre for Agriculture and the Bioeconomy (QUT). The shoot and root rice samples were dried in a hot air oven at 70 °C for three days and freeze-dried at −80 °C for 24 h using Virtis Benchtop Pro freeze dryers. The freeze-dried sample was then ground to fine powder using Tissue Lyser II. Approximately 0.2–0.3 g of freeze-dried sample was weighed using an analytical balance and transferred to a 50 mL Falcon tube. Samples were pre-digested with acid mixtures (3 mL HNO_3_ 70% and 1 mL HCl 40%) and mixed well by vortexing and left overnight in a fume hood at room temperature. On the following morning, the sample was digested at 80 °C for 30 min followed by 2 h at 125 °C in a dry heat block. After the digestion, the sample was cooled to room temperature before diluting with 22 mL of distilled water to make a final volume of 25 mL. The tube containing samples was centrifuged at 4500 rpm for 10 min, and 10 mL of supernatant was transferred to a polypropylene tube. Element content analyses were carried out using Perkin Elmer Optima 8300 DV Inductively Coupled Plasma Optical Emission Spectrometer (ICP-OES) following the manufacturer’s instructions. Data were analyzed using Syngistix software V2.0.

### 4.8. Measurement of Electrolyte Leakage

Electrolyte leakage, during salinity stress assay, was measured using a CM 100-2 conductivity meter (Reid & Associates CC, DU, South Africa) following the manufacturer’s instructions. A 0.5 cm piece of the second youngest fully expanded leaf was briefly excised and placed inside a zip-lock plastic bag and immediately put on ice. Then, the leaf piece was rinsed with deionized water and loaded into wells of the CM 100-2 conductivity meter containing 1.25 mL of deionized water. Measurement was carried out every two min over 60 min periods. Following the measurement, samples were dried in an oven at 70 °C overnight, then weighed (dry weight). Electrolyte leakage was calculated as the slope of electrolyte leakage over time and normalized by dry weight.

### 4.9. Statistical Analysis

The salinity screening experiments were designed in a completely randomized block design with three replications. One-way Analysis of Variance (ANOVA) analyzed the statistical significance of mean values with Turkey HSD multiple comparisons with three replicates (MiniTab version 17, Sydney, NSW, Australia).

## Data Availability

The authors confirm that the data supporting the findings of this study are available within the article.

## Supplementary Materials

The following are available online at www.mdpi.com/article/10.3390/plants10102044/s1, Table S1: Plant materials, Table S2: Primers used in this study.
