# Supplementary material for: Rapid Accumulation of Proline Enhances Salinity Tolerance in Australian Wild Rice Oryza australiensis Domin
Source: Plants (Basel). 2021 Sep 28;10(10):2044. doi: 10.3390/plants10102044 (PMC8540606; doi:10.3390/plants10102044)
Supplement: Supplementary file 1 [file plants-10-02044-s001.zip › plants-1370168-supplementary.pdf]

plants

Article

Rapid Accumulation of Proline Enhances Salinity Tolerance in Australian Wild Rice *Oryza australiensis* Domin.

Ha Thi Thuy Nguyen [1,*], Sudipta Das Bhowmik [1], Hao Long [1], Yen Cheng[1], Sagadevan Mundree [1], and Linh Thi My Hoang [1,*]

Supplementary Materials

Supplemental Table S1. Plant materials used in this study

AGG Accession number	Primary accession name	Taxonomy	Origin state	Latitude	Collection site details
300130	JC 2304	*Oryza australiensis* Domin	North Territory	-19.9983	25.4 km W of QLD/NT border, approx 1.5 km W of cattle creek
	Pokkali	*Oryza sativa* L.	Queensland		CAB laboratory, QUT
	Nipponbare	*Oryza sativa* L.	Queensland		CAB laboratory, QUT

Supplemental Table S2. Primers used in this study

Gene	Forward primer	Amplicon length (bp)	Ref.
OsP5CS1	GATTGGGTGCTGAGGTTGGCATAA<br>CGACATCCTTGTCACCATTCACCA	132	[76]
OsP5CS2	AAATTGTCGTCTGAGGAGCGT<br>GGTCATCCGAGCAACCAATGA	150	
OsP5CR	TCCGGGTCAGCTGAAGGATA<br>GAAAGTTCACGGCAACGCTT	138	
OsProDH	GTGAGCAAGTACCTGCCGTA<br>TTGCAGCCTTGAACCTCCTC	148	
OsActin	CTGCGGGTATCCATGAGACT<br>GCAATGCCAGGGAACATAGT	118	[76]
OsEF1-α	GCTGGTGACCAAGATCGACA<br>TGGGCTTGGTGGGAATCATC	107	
